# A validation study of an esophageal probe–based polygraph against polysomnography in obstructive sleep apnea

**DOI:** 10.1007/s11325-021-02374-4

**Published:** 2021-06-28

**Authors:** Thorarinn Arnar Olafsson, Eivind Andreas Steinsvik, Gregor Bachmann-Harildstad, Harald Hrubos-Strøm

**Affiliations:** 1 Department of Otorhinolaryngology, Akershus University Hospital, PO 1000 1470 Lørenskog, Norway; 2 Faculty of Clinical Medicine, University of Oslo, Oslo, Norway; 3 Faculty of Basic Medical Sciences, University of Oslo, Oslo, Norway

**Keywords:** Obstructive sleep apnea, Polysomnography, Oximetry, Home sleep apnea testing, Validation, Sleep disordered breathing

## Abstract

**Study objectives:**

The aim of this study was to validate the automatically scored results of an esophageal probe–based polygraph system (ApneaGraph**®** Spiro) against manually scored polysomnography (Nox A1, PSG) results. We compared the apnea–hypopnea index, oxygen saturation index, and respiratory disturbance index of the devices.

**Methods:**

Consenting patients, referred for obstructive sleep apnea workup, were tested simultaneously with the ApneaGraph**®** Spiro and Nox A1**®** polysomnograph. Each participant made one set of simultaneous registrations for one night. PSG results were scored independently. Apnea–hypopnea index, oxygen desaturation index, and respiratory disturbance index were compared using Pearson’s correlation and scatter plots. Sensitivity, specificity, and positive likelihood ratio of all indices at 5, 15, and 30 were calculated.

**Results:**

A total of 83 participants had successful registrations. The apnea–hypopnea index showed sensitivity of 0.83, specificity of 0.95, and a positive likelihood ratio of 5.11 at an index cutoff of 15. At a cutoff of 30, the positive likelihood ratio rose to 31.43. The respiratory disturbance index showed high sensitivity (> 0.9) at all cutoffs, but specificity was below 0.5 at all cutoffs. Scatterplots revealed overestimation in mild OSA and underestimation in severe OSA for all three indices.

**Conclusions:**

The ApneaGraph**®** Spiro performed acceptably when OSA was defined by an AHI of 15. The equipment overestimated mild OSA and underestimated severe OSA, compared to the PSG.

## Introduction

Manually scored polysomnography (PSG) is considered the most comprehensive method for the workup of sleep disorders [1]. It is, however, a cumbersome method that registers a multitude of parameters. This has led to the development of smaller polygraphs or home sleep apnea testing (HSAT) devices that monitor fewer variables but are easier to use. Ideally, a sleep polygraph has some way of measuring respiratory effort, flow through the airway, and blood oxygen saturation [2, 3]. These devices cannot directly detect the states of sleep or wakefulness, as they do not register an electro-encephalogram, electro-myelography, and electro-oculography for sleep staging. However, lately, several HSAT do indirectly estimate sleep and wakefulness using available parameters.

Concerning the measurement of airflow, the American Academy of Sleep Medicine (AASM) has, since 2007, recommended use of both nasal cannula and thermistor for the scoring of obstructive apneas and hypopneas [4]. Most HSAT devices use either a nasal cannula or a thermistor-based flow assessment.

An estimate of respiratory effort is important in order to establish whether reduced airflow is due to obstruction or simply decreased respiratory effort. When measuring respiratory effort, manometry by esophageal probe was previously considered the gold standard [5, 6]. The esophageal probe has an added possibility of measuring differential airflow through the nose or the pharynx, providing information on the location of the obstruction [7].

While efforts have been made to standardize the endpoints of sleep workup, there is an ongoing debate concerning which outcome measures are relevant [8–11]. The most common standards are the apnea–hypopnea index (AHI) and the oxygen desaturation index (ODI). These indices do not factor in arousals independently, and there is some debate on whether a subset of patients will go undiagnosed if arousals related to minor respiratory events are not scored [3]. The respiratory disturbance index (RDI) is less commonly used, but a more sensitive endpoint [12]. It factors in apneas, hypopneas, and respiratory event–related arousals (RERAs) [1].

The ApneaGraph® Spiro is a HSAT device that uses an esophageal probe to measure air flow and respiratory effort. An accompanying software package scores the results automatically and provides output in the form of the main indices, AHI and ODI, and estimates the RDI from available data.

The aim of this study was to validate the automatically scored results of an esophageal probe–based polygraph system against manually scored polysomnography results. We compared the AHI, ODI, and RDI of the devices.

## Materials and methods

**Fig. 1:**
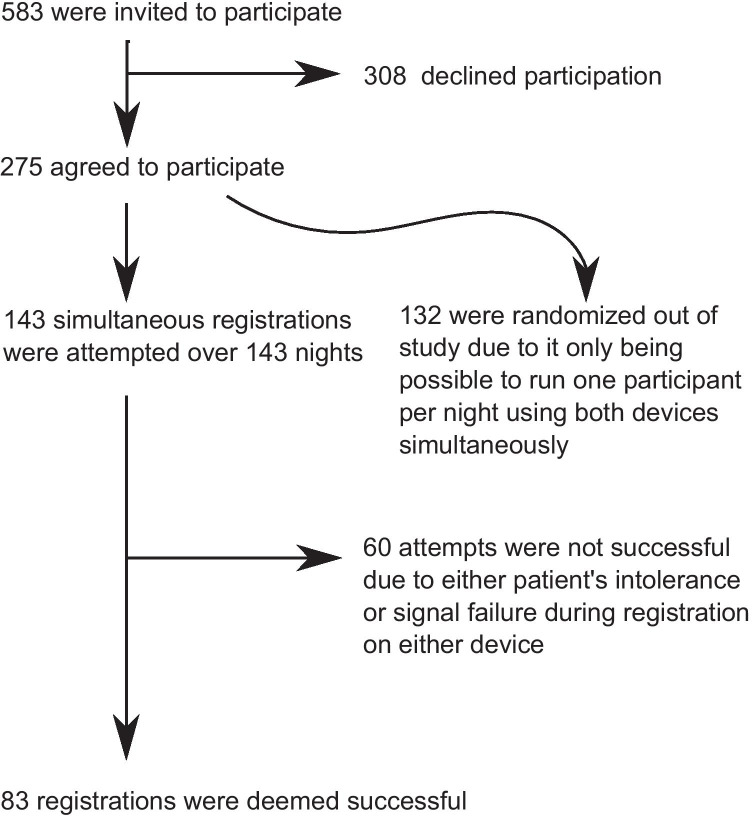
Flowchart of recruitment process

Participants were recruited from patients referred to the Akershus University Hospital for workup of sleep disordered breathing. Patients with acute systemic disease, age under 18, poor understanding of the Norwegian language, and prior or current treatment for OSA were excluded. Consenting participants were mounted simultaneously with a polysomnograph and the ApneaGraph® Spiro and slept overnight at the clinic. Only one patient could be registered per night, and if multiple patients were willing to participate, one was selected using a random number generator. Figure 1 illustrates the recruitment process. We did not record the number of participants, who specifically refused to accept an esophageal probe. Non-participants were mostly registered with another catheter-based HSAT, the Apneagraph 200.

The PSG results were used for clinical purposes and reviewed the morning after. The ApneaGraph® Spiro data were downloaded in batches and reviewed. This resulted in a period of failed Apneagraph@ Spiro studies. This was corrected with a service check of the device.

The PSG used was the A1, by Nox Medical, Reykjavik, Iceland. It registered an eight-lead electroencephalography, electromyography of the chin and leg, electro-oculography, pulse oximetry (probe and sensor by Nonin, sampling rate 75 Hz, 1-s average for each pulse beat), nasal flowmetry via cannula, ECG, RIP belts on chest and abdomen, microphone on chest, actimetry, and positional registration. PSG results were scored by a single, independent sleep technician using the Noxturnal interface. The American Academy of Sleep Medicine (AASM) 2012b criteria were used for scoring. A 4% desaturation threshold was used when scoring hypopneas [13]. In addition, respiratory event–related arousals were scored, and an RDI was calculated, defined as AHI + RERA index.

**Fig. 2:**
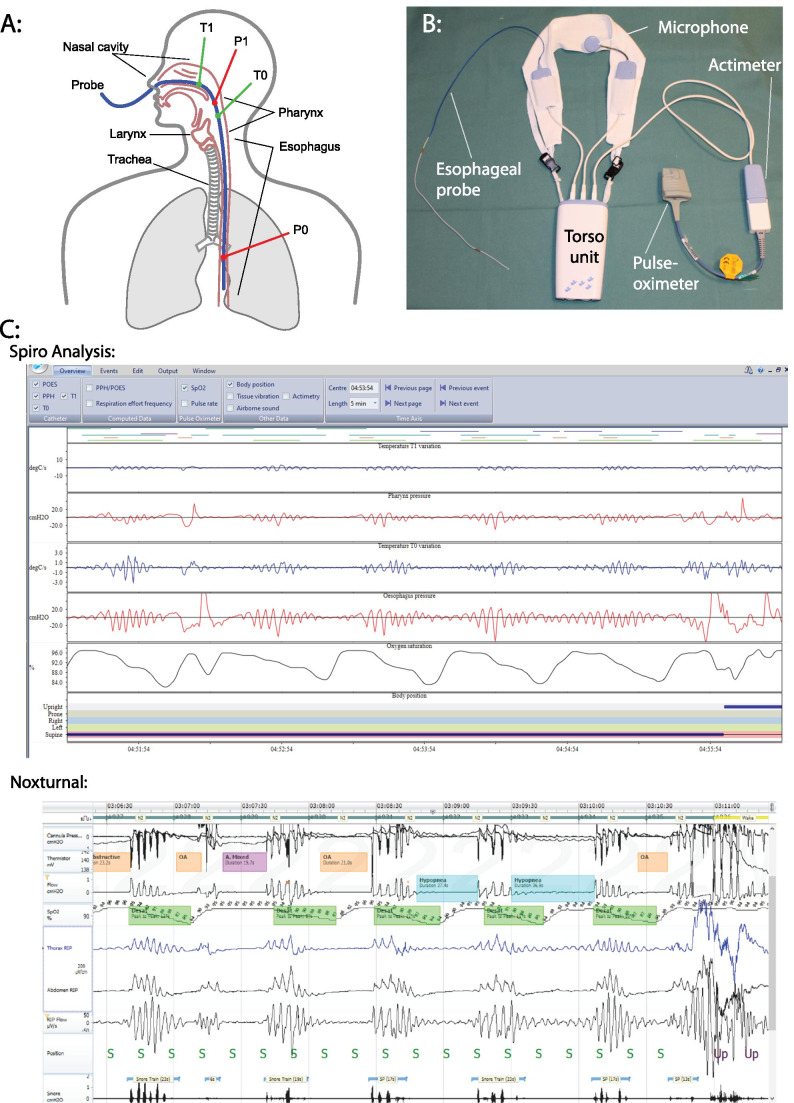
The ApneaGraph and Spiro Analysis. **a** A schematic illustration showing the placement of the esophageal probe and its sensors. **b** The torso unit, arm unit with pulse oximeter, actimeter, microphone, and esophageal probe. **c** A 5-min epoch showing several obstructive events and an arousal, as seen in by the ApneaGraph in Spiro Analysis 3.2 (top) and the PSG registration as seen in Noxturnal

The ApneaGraph® Spiro (by Spiro Medical, Bergen, Norway) is shown in Fig. 2b. The probe has two pressure meters placed in the pharynx and esophagus to evaluate respiratory effort (Fig. 2a). It also uses two thermistors placed in the nose and pharynx to evaluate the flow. The ApneaGraph® Spiro also registers pulse oximetry (sensor by Smiths Medical, UK; probe by Metko Ltd., Turkey, sampling rate 60 Hz, average over two pulses), activity and position of the torso unit, activity of the arm probe, and snoring. This was registered with a microphone taped to the throat. The device was mounted by a sleep technician. The esophageal probe was positioned trans-nasally, using a lidocaine gel. The software accompanying the ApneaGraph® Spiro, called Spiro Analysis (v. 3.2), used a default 4% desaturation threshold for apneas, and 30% flow reduction for hypopnea and RERAs. RERAs were defined as 10-s flow limitation and increased respiratory effort, followed by release of effort and normalization of flow [14]. The registration is available for review in the software (Fig. 2c), where events can be manually registered or altered. For this study, the automated results were used. Indices are calculated using estimated sleep time. The Spiro Analysis software estimates the state of wakefulness or sleep by interpreting events from actimetry data on the arm probe, position and rotation of the torso unit, snoring, swallowing events, hypopneas, apneas, and RERAs. An event density is calculated for each parameter in an epoch. The software then estimates the state of sleep or wake in 5-min epochs, using a weighted sum of event densities in the epoch.

A minimum of 4 h of continuous registration on both devices with acceptable signal quality was defined as successful.

For clarity in this article, corresponding indices are referred to by corresponding names, as defined in Table 1.

**Table 1:** Definitions of indices

Index acronym	Definition of index	
AHI_psg_	A + H	/hour of sleep registered on PSG
ODI_psg_	Number of desaturations	/hour of sleep registered on PSG
RDI_psg_	A + H + RERA	/hour of sleep registered on PSG
AHI_spiro_	A + H	/hour of estimated sleep time on ApneaGraph**®**
ODI_spiro_	Number of desaturations	/hour of estimated sleep time on ApneaGraph**®**
RDI_spiro_	A + H + RERA	/hour of estimated sleep time on ApneaGraph**®**

*A*, number of obstructive apneas; *H*, number of obstructive hypopneas; *RERA*, number of respiratory event–related arousals

### Statistical analysis

Statistics were calculated using SPSS (by IBM) and Excel (by Microsoft). Scores were compared using two-tailed Pearson’s test. Data are presented in *x* = *y* scatter plots. The standard disease-defining cutoffs of 5, 10, and 30 were used to calculate sensitivity, specificity, and receiver operating characteristics (ROC) curves for each index.

In all instances, each index from the ApneaGraph® Spiro was compared directly to, and only to, its counterpart on the PSG, i.e., AHI_spiro_ to AHI_psg_, ODI_spiro_ to ODI_psg_, and RDI_spiro_ to RDI_psg_.

## Results

A basic description of the study population is displayed in Table 2. In total, 60 attempts at double registration were unsuccessful (Fig. 1). Fourteen registrations were lost when a critical failure of the ApneaGraph® Spiro went unnoticed for a period. Otherwise, 20 ApneaGraph® recordings were unsatisfactory, due to loss of either esophageal probe data or SpO2 signal. There were eleven cases that resulted in no ApneaGraph® recordings and only a PSG, due to refusal of patient after consent or problems mounting the device. A total of 21 registrations were lost due to unsatisfactory PSG registration. Six of the 32 cases with low quality had unsatisfactory signal in both devices. No significant difference was found between participants with double registration and those that could not participate due to randomization (26% female, mean BMI 31.4). The same was true for those 60 participants who attempted registration, but were not successful (24% female, mean BMI 29.1).

**Table 2:** Descriptive data of study population

	Male	Female	Total
	Mean (SD, range)	Mean (SD, range)	Mean (SD, range)
Age	45.0 (10.6, 26–77)	45.4 (10.6, 22–62)	45.1 (10.5, 22–77)
BMI	30.3 (5.6, 21.7–50.5)	31.3 (6.7, 19.5–44.6)	30.6 (5.9, 19.5–50.5)
SBP	136 (15.4, 107–190)	134 (16.6, 110–184)	136 (15.7, 107–190)
DBP	79 (10.1, 51–104)	74 (8.3, 59–90)	77.8 (9.9, 51–104)
Epworth score	10.3 (4.3, 0–19)	11.9 (4.9, 1–18)	10.5 (4.5, 0–19)
AHI_psg_	30.6 (27.5, 0.5–105)	12.6 (16.1, 0.2–67)	25.6 (26.1, 0.2–105)
AHI_spiro_	22.1 (18.2, 1.9–75)	9.6 (7.4, 2.3–28)	18.7 (16.8, 1.9–74)
ODI_psg_	26.2 (26.2, 0.3–93)	10.9 (16.0, 0.1–67)	22.0 (24.7, 0.1–93)
ODI_spiro_	28.8 (20.7, 2.3–89)	15.2 (9.9, 3–35)	25.0 (19.3, 2.3–89)
RDI_psg_	36.4 (28.3, 0.5–107)	15.9 (18.4, 0.5–77)	30.7 (27.4, 0.5–107)
RDI_spiro_	47.7 (18.9, 6.3–93)	31.9 (13.6, 8–63)	43.3 (18.9, 6.3–93)
Number of participants, % of total	60, 73%	23, 27%	83, 100%

### Correlation of indices

**Fig. 3:**
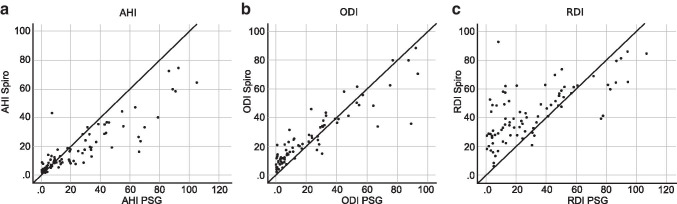
Scatter plots with a 45° line representing perfect correlation. **a** AHI_spiro_ vs AHI_psg_. **b** ODI_spiro_ vs ODI_psg_. **c** RDI_spiro_ vs RDI_psg_

The AHI_spiro_ showed a Pearson’s correlation of *r* = 0.884 to the AHI_psg_. The ODI showed a correlation of *r* = 0.909 between devices. The RDI_spiro_ showed a correlation of *r* = 0.685 to the RDI_psg_. Figure 3 shows the scatter plots comparing the different indices.

Bland–Altman plots (not shown) also confirmed a tendency of overscoring in the lower range and under-scoring with increasing indices.

### Classification of OSA severity

**Fig. 4:**
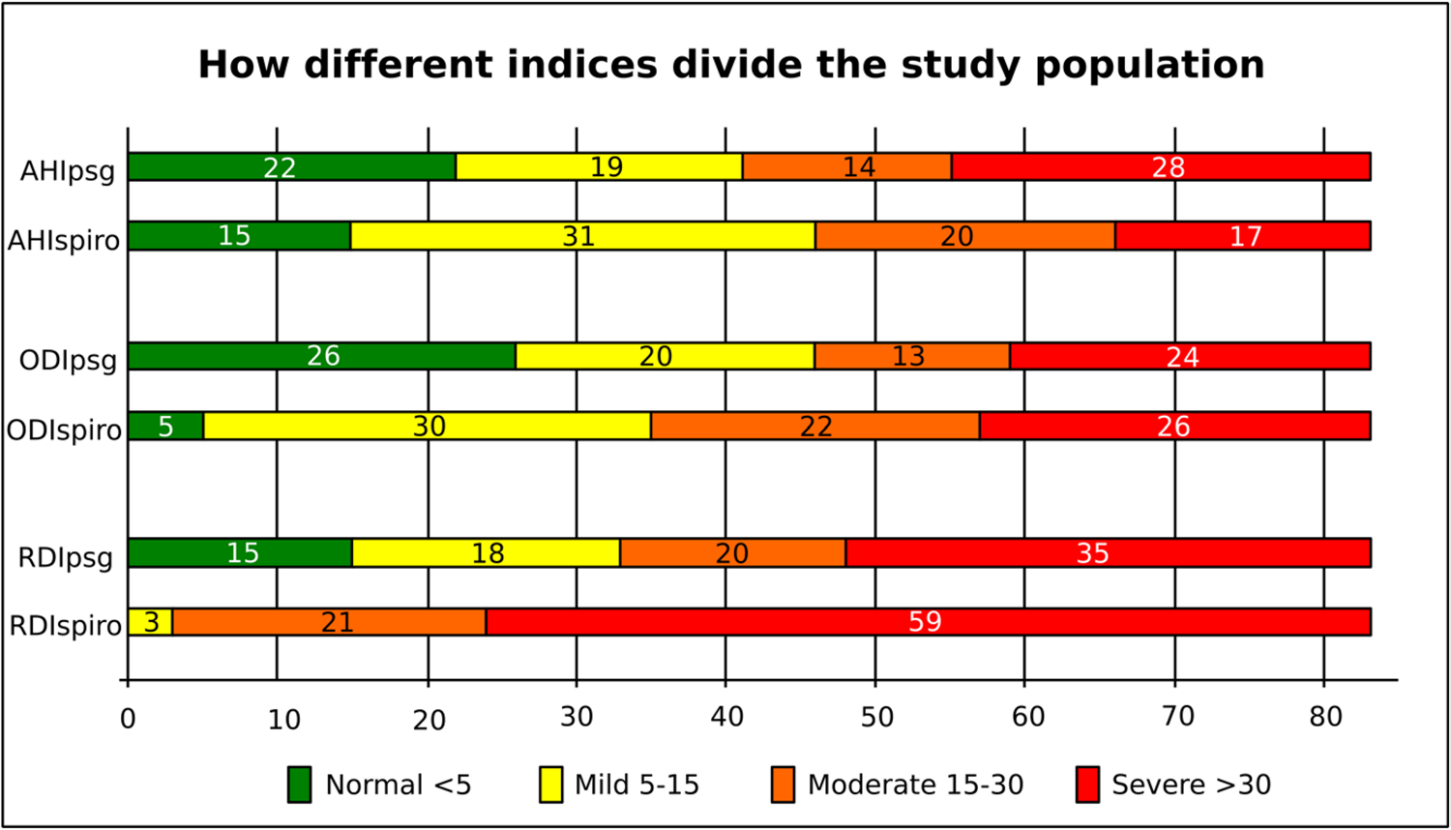
Diagram showing how different indices divided the study population according to OSA severity

Using the standard index cutoffs of 5, 15, and 30, the AHI divides the population into four groups, those with no OSA, mild OSA, moderate OSA, and severe OSA (Fig. 4).

The AHI_psg_ split the population: 22 with no disease (AHI < 5), 19 with mild disease (AHI 5–15), 14 with moderate disease (AHI 15–30), and 28 with severe disease (AHI > 30).

The AHI_spiro_ split the population: 15 with no disease (AHI < 5), 31 with mild disease (AHI 5–15), 20 with moderate disease (AHI 15–30), and 17 with severe disease (AHI > 30).

### Diagnostic values

Sensitivity and specificity ratios are depicted in Table 3. The diseased state can be redefined using different cutoffs on the indices generated by the PSG. Sensitivity and specificity for each cutoff were calculated for every index of the ApneaGraph® Spiro. AHI, ODI, and RDI were all sensitive (> 0.80) at all cutoffs, except AHI at 30, showing only sensitivity of 0.57. All indices showed poor specificity at a cutoff of 5. The AHI showed good specificity (> 0.95) at 15 and 30. The RDI showed poor specificity at all cutoffs.

**Table 3:** Diagnostic values of the ApneaGraph® Spiro

Disease-defining cutoff	Index	Sensitivity	95% CI	Specificity	95% CI	Positive likelihood ratio	Area under the ROC curve
5	AHI_spiro_	1.00	n-a	0.68	(0.49–0,88)	2.75	0.96
	ODI_spiro_	1.00	n-a	0.19	(0.04–0.34)	1.24	0.87
	RDI_spiro_	1.00	n-a	0.00	n-a	1	0.82
15	AHI_spiro_	0.83	(0.72–0.95)	0.95	(0.89–1.02)	5.11	0.93
	ODI_spiro_	0.97	(0.91–1.02)	0.74	(0.61–0.87)	3.73	0.90
	RDI_spiro_	1.00	n-a	0.09	(0.00–0.19)	1.10	0.76
30	AHI_spiro_	0.57	(0.39–0.75)	0.98	(0.95–1.02)	31.43	0.96
	ODI_spiro_	0.96	(0.88–1.04)	0.95	(0.89–0.98)	18.85	0.92
	RDI_spiro_	0.91	(0.82–1.01)	0.44	(0.30–0.58)	1.62	0.83

Sensitivity, specificity, positive likelihood ratio, and areas under ROC curve of all indices of the ApneaGraph® Spiro, when compared to their counterpart on the PSG, at different disease-defining cutoffs

*CI*, confidence interval; *n-a*, not applicable

**Fig. 5:**
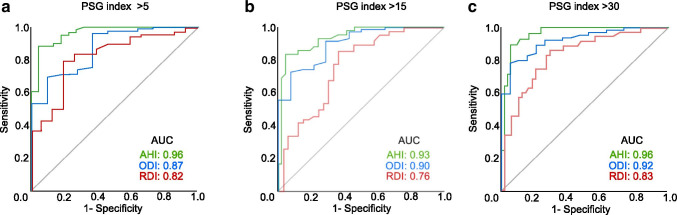
ROC curves for all ApneaGraph**®** Spiro indices. The study population was split into healthy and diseased groups as based on the standard cutoffs on the corresponding PSG index. **a** 5. **b** 15. **c** 30. AUC, area under curve

Receiver operating characteristic (ROC) curves give a graphic representation of sensitivity and specificity of the device being tested against an accepted standard (Fig. 5). The areas under the curves were consistently highest for the AHI with values of 0.93 or higher at all cutoffs (Table 3).

## Discussion

### Main findings

The AHI and ODI results of the Apneapraph® Spiro compared reasonably to the AHI and ODI of the PSG. The RDI of the ApneaGraph® Spiro deviated from the RDI of the PSG.

### Apnea–hypopnea index

Different technologies calculate indices, such as the AHI, in different ways based on different parameters. Regardless of the device used, it is important that the indices are comparable between different devices. The AHI of the ApneaGraph® Spiro underscored compared to the PSG in higher ranges, and overscored in the low range. In general, it was useful in classifying the OSA severity of patients. The AHI_spiro_ was sensitive at all cutoffs but yielded a number of false positives at lower values. Collop et al. [15] proposed that the AHI of a polygraph should show a sensitivity of over 0.825, and a positive likelihood ratio over 5. The AHI_spiro_ meets these criteria for the diagnosis of moderate OSA, but not for mild disease or severe disease. In the mild cases, this is due to several false positives in the low range yielding a positive likelihood ratio of 2.75, although sensitivity was 100%. In severe cases, the ApneaGraph® Spiro underscored somewhat, resulting in lower sensitivity, although the positive likelihood ratio was high.

The ROC curve for the AHI cutoff at 5 showed a good result, with an area under the curve of 0.96, indicating that a slight shift in cutoff value would have improved the positive likelihood ratio.

The AHI of the ApneaGraph® Spiro seems to perform reasonably compared to other HSAT devices [16–20] that have been validated. A general criticism of HSAT devices has been their lack of sensitivity compared to PSG [3, 21], but in contrast, the ApneaGraph® Spiro that was sensitive to mild disease and skewed more towards generating false positives — rather than false-negative results.

### Oxygen desaturation index

The ODIs were expected to be identical between devices, and indeed, the ODI_spiro_ showed the highest Pearson correlation of the three indices at 0.909. There was, however, a surprising systemic error with consistent overscoring of the ApneaGraph® Spiro in the low range. This resulted in a high number of false positives and poor specificity of the ODI at cutoff of 5. The lower range is the range in which the clinical severity of disease is estimated, and difference of outcome can impact the choice of treatment. For example, an index variation of 5 vs 15 can have immediate implications for the choice of treatment, whereas variation of 35 vs 45 has few implications as in both cases immediate treatment is indicated [22, 23]. This discrepancy can be due to a difference in pulse oximetry specifications, which is, regrettably, not completely standardized for the purposes of sleep workup. Both devices exceed the minimum criteria placed by AASM with regards to sampling rate over 10 Hz and sample averaging over less than 3 s [4], but they do so in different ways. The ApneaGraph® Spiro samples at 60 Hz and average samples over two pulse beats, while the A1 samples at 75 Hz and averages samples 1 s prior to each pulse beat. This discrepancy possibly underlines a need for a further standardization of pulse oximetry devices, since this is a critical variable that underpins the AHI, ODI, and RDI.

### Respiratory disturbance index and respiratory effort

There has been some lack of clarity regarding RERAs and the RDI, with only a limited subset of the literature on sleep disordered breathing reporting either RERA or RDI, as reported by Krakow et al. [1, 12].

A question can be raised on the relevance of the RDI. Its aim is to identify patients not only with OSA but also with milder symptoms of disordered breathing. In 1982, this was already defined as upper airway resistance syndrome [24, 25]. The term did not get mainstream attraction, as focus was on the more severe OSA syndrome. The change of AASM scoring rules in 2012, from desaturation thresholds for hypopneas of 4 to 3%, made the AHI more sensitive, as it now scored many events, only the RDI would have registered otherwise. There are, nonetheless, events an RDI would include, that an AHI scored at 3% desaturation limit would not [1].

In this study, the difference between the RDI indices from the two devices is difficult to interpret. Comparing them is somewhat like comparing apples and oranges, since they register different events. The RERA of the PSG is scored from a 10-s flow limitation of the nasal cannula and accompanying arousal as defined on EEG, while on the ApneaGraph**®** Spiro a 10-s flow limitation ending in increased activity. It is worth noting that the EEG represents cortical brain activity and does not register increased activity in the lower levels of the brain [26]. Considering that respiratory reflexes are subcortical and can induce a change in breathing patterns and muscle tonus without affecting the cortex, the ApneaGraph® Spiro will register different respiratory events than the PSG. Both variants of obstruction-related change in breathing patterns could be considered a disturbance of sleep and may contribute to health impairment. Finally, there are few reports on the characteristics of HSAT devices that try to estimate the RDI. The other HSAT devices, that claim to be able to estimate RERAs, report a better concordance with PSG results than seen here [18, 27]. Obrien [27] reported considerably better concordance of RDI indices between a peripheral arterial plethysmography device and PSG. However, results were presented using ROC curves, and sensitivity and specificity were not reported for all standard cutoffs making comparison somewhat difficult.

### The effects of an esophageal probe on PSG results

The presence of an esophageal device could potentially affect the results of a PSG. The probe could act as a splint that directly maintains an open airway where it would otherwise collapse. On the contrary, a catheter takes up space in an already stenotic airway and potentially causes obstruction. Maddison et al., who addressed this directly, found that a thin esophageal probe does not alter the airway collapsibility during propofol anesthesia [28]. On the contrary, Virkkula and co-workers reported that the ipsilateral nasal resistance was clearly elevated when measured with a catheter used overnight compared with the control measurement [29].

The probe also provides a mechanical stimulus to the nasal and pharyngeal mucosa that could affect arousal level and respiratory drive during natural sleep. Chervin and Aldridge studied the effect of esophageal manometry on sleep architecture, using waterfilled catheters, substantially thicker than the transistor-based probes available today. Their conclusion was that esophageal manometry does not significantly affect sleep architecture as measured using a PSG [30].

### Disease-defining cutoffs

The disease-defining index cutoffs of 5, 15, and 30 are the traditional measures of OSA severity, and they have been used in sleep research for a long time.

They are, however, not based on clinical evidence, but are rather approximate numbers decided upon in the early days of sleep research. They have been used unchanged in the literature, even though the rules for scoring hypopneas have changed. Lately, it has been discussed to revise the disease-defining cutoffs based on longitudinal outcomes of OSA patients [31]. It is worth noting that a minor change in the disease-defining cutoffs would significantly affect all results in this study.

It is also debatable whether the same cutoffs are applicable for all indices, as the 5, 15, and 30 cutoffs are originally defined for AHI, but have since been used when evaluating both ODI and RDI, as discussed above. They are useful for comparison of the indices, even if their exact clinical relevance is debatable.

### Strengths and limitations

The main strength of this study is its sample size compared to validation studies of other catheter-based devices [21, 23].

In-lab registration is a relative weakness of the study design. The purpose of the study was, however, to do time-synchronized, simultaneous measurements in a controlled environment*.*

A clear weakness of the study was the high number of subjects that were not able to participate or had a failure of registration.

Regarding the latter, registrations were lost due to failure of both the ApneaGraph® Spiro and the A1 PSG. A weak point of the PSG was mainly a loss of the oximetry signal, while on the ApneaGraph®, the esophageal probe signals failed. On the other hand, the low success rate in our university hospital sponsored study provides data from a real-world setting. Considering that validation studies of HSAT devices are not numerous, there may exist a publication bias in this regard.

The lack of data on reasons for non-participation is a limitation. However, because non-participants were also diagnosed with a catheter-based HSAT, we believe other factors than the use of an endonasal catheter explained most of this rate. For example, participation involved being monitored by two devices during the night and completing extra questionnaires. There was also no financial incentive or preferential treatment offered to patients who participated.

## Conclusion

The AHI and the ODI of the ApneaGraph**®** Spiro can be used for detecting moderate and severe OSA in a population with high prevalence of sleep disordered breathing. However, there was poor correlation between the RDI of the PSG and that of the ApneaGraph**®** Spiro.

Further standardization of pulse oximetry in sleep workup might be considered.

Finally, longitudinal studies should be performed to determine predictive properties of different indices on hard outcomes.
